# Exploring HERV-K (HML-2) Influence in Cancer and Prospects for Therapeutic Interventions

**DOI:** 10.3390/ijms241914631

**Published:** 2023-09-27

**Authors:** Bárbara Costa, Nuno Vale

**Affiliations:** 1 OncoPharma Research Group, Center for Health Technology and Services Research (CINTESIS), Rua Doutor Plácido da Costa, s/n, 4200-450 Porto, Portugal; b.c.211297@gmail.com; 2 CINTESIS@RISE, Faculty of Medicine, University of Porto, Alameda Professor Hernâni Monteiro, 4200-319 Porto, Portugal; 3 Department of Community Medicine, Information and Health Decision Sciences (MEDCIDS), Faculty of Medicine, University of Porto, Rua Doutor Plácido da Costa, s/n, 4200-450 Porto, Portugal

**Keywords:** human endogenous retrovirus, cancer, HERV-K (HML-2), viral infection, antiviral drugs

## Abstract

This review investigates the intricate role of human endogenous retroviruses (HERVs) in cancer development and progression, explicitly focusing on HERV-K (HML-2). This paper sheds light on the latest research advancements and potential treatment strategies by examining the historical context of HERVs and their involvement in critical biological processes such as embryonic development, immune response, and disease progression. This review covers computational modeling for drug-target binding assessment, systems biology modeling for simulating HERV-K viral cargo dynamics, and using antiviral drugs to combat HERV-induced diseases. The findings presented in this review contribute to our understanding of HERV-mediated disease mechanisms and provide insights into future therapeutic approaches. They emphasize why HERV-K holds significant promise as a biomarker and a target.

## 1. Retrovirus: Human Endogenous Retroviruses (HERVs)

Human endogenous retroviruses (HERVs) are remnants of ancient retroviral infections that were integrated into the human genome millions of years ago [1]. These viral elements comprise approximately 8% of the human genome [2]. Initially considered as “junk” DNA with no apparent function, research in recent years has revealed their intriguing role in human health and disease [3,4,5,6]. There are numerous endogenous retrovirus-like sequences in the human genome. A human haploid genome contains 30–50 members of the human endogenous retrovirus type K (HERV-K) family, which is also substantially conserved in Old World monkeys and apes [7]. While most HERVs have accumulated mutations that have compromised their coding capacity, some retain intact open reading frames (ORFs) [8,9]. HERVs have been found to influence various biological processes, including embryonic development, immune response, and even the development of certain diseases [4]. The most recently integrated and best conserved human proviruses are found in the HERV-K family (HML-2) [10]. The HERV-K group can be divided into ten families [11,12]. The term “K” comes from their use of a lysine tRNA to promote reverse transcription [13,14], and “HML-2” indicates their relationship with the murine betaretrovirus mouse mammary tumor virus (MMTV) [15,16,17]. 

Endogenous retroviruses are present as proviruses, the integrated state of retroviral DNA within the host’s germ-line DNA, passed from one generation to another [18]. The integration of additional germ-line proviruses has been shown to modify the arrangement and activity of cellular genes [19,20]. Over 90 proviruses from the extraordinarily well-preserved HML-2 family still contain the ORFs that code for functional viral proteins. While the ability of endogenous retroviruses to replicate can lead to potential illness, they can also shield the host against diseases induced by retroviruses [20,21,22,23,24]. Endogenous retroviruses derive from exogenous retroviruses. Exogenous retroviruses are horizontally transmitted between infected and uninfected hosts, being stably integrated with the genome of the host species from which they derive [25]. HERVs have a similar genetic sequence to exogenous retroviruses [26]. A complete sequence of HERVs comprises gag, pol, pro, and env regions packed between two long terminal repeats (LTRs), acting as promoter sequences [27,28]. The gag gene codes for structural components of the matrix (MA), capsid (CA), and nucleocapsid (NC). The pol gene codes for integrase, reverse transcriptase, and RNAse. The pro gene codes for the protease, and the env gene encodes the env glycoprotein that comprises surface subunit (SU) and transmembrane (TM) subunits [4,8,29]. In terms of taxonomy, HERVs can be categorized into three groups based on their similarity in evolutionary relationships: Class I includes Gammaretrovirus and Epsilonretrovirus; Class II encompasses Deltaretrovirus, Alpharetrovirus, Lentivirus, and Betaretrovirus; and Class III comprises Spumaretrovirus [4,30]. Examples of exogenous retroviruses of clinical relevance are the human immunodeficiency viruses (HIV-1, HIV-2) and human T-lymphotropic viruses (HTLV-1, HTLV-2) since they are associated with diseases like AIDS and various forms of leukemia [31]. 

The presence of endogenous retroviruses in the human genome can positively and negatively affect the host. On the positive side, endogenous retroviruses have been shown to play a role in embryonic development, placenta formation, and immune response regulation. During early human embryogenesis, HERVs are systematically transcribed stage-specific, and their expression serves as a hallmark of cellular identity and potency in early human embryos [25,32]. This hallmark of cellular identity is also retained in mature cells [33]. Specifically, HERV-K (HML-2), the most recently active endogenous retrovirus group in humans, is transcribed during normal human embryogenesis, starting from the eight-cell stage and continuing through the emergence of epiblast cells in preimplantation blastocysts. These proviral RNAs produce viral-like particles and gag proteins in human blastocysts, indicating the presence of retroviral products during early human development [34,35]. Additionally, the envelope protein of HERV-K (HML-2) from specific loci in chromosomes 12 and 19 is highly expressed on the cell membrane of human pluripotent stem cells (hPSCs) [36]. It is critical in maintaining stemness through the mammalian target of the rapamycin (mTOR) pathway [37,38]. 

On the negative side, endogenous retroviruses can have pathogenic potential. Unlike most other HERVs, ORFs encoding retroviral proteins remained in HERV-K [9]. Except for some pathological situations such as germ-cell tumors, melanoma, and HIV infection, the host silences HERV-K transcriptionally [10]. Understanding the impact of HERVs on human biology has opened new avenues of research and potential therapeutic interventions [11]. A minority of the global population has recently been discovered to possess what appears to be an undamaged provirus, suggesting a relatively recent acquisition. Furthermore, specific lineages, including the HERV-K (HML-2) group, have been confirmed to produce viral particles [11,12].

The downregulation or epigenetic silencing of HML-2 env results in the dissociation of stem cell colonies and enhanced differentiation along neuronal pathways, suggesting the significance of HML-2 regulation in human embryonic development and neural differentiation [38]. A delicate balance between endogenous retrovirus activation and repression exists throughout pre-implantation embryo development, and a temporal window for endogenous retrovirus expression is created due to the remodeling of heterochromatic markers and relaxed chromatin structure [39]. Zygotic genome activation (ZGA) is a critical event in pre-implantation embryo development, where the paternal genome undergoes genome-wide loss of DNA methylation, and H3K4me3 and H4 acetylation occur, leading to transcriptional activation and translational activity during major ZGA—failures in ZGA result in developmental arrest and inability to progress further [40]. Therefore, HERVs are involved in early development by reshaping the gene regulatory network of the preimplantation embryo [41], and their expression is associated with undifferentiated states, phenotypic plasticity, and stem cell characteristics [42], which are traits linked to aggressive cancer and poor patient outcomes. While HERV expression is tightly controlled in normal adult tissues, it is reported to be abnormally expressed in various diseases, including cancer, inflammatory, neurological, aging, and viral infections. 

HERVs exhibit a range of significant functions. Firstly, they can serve as promoters or enhancers of antiviral genes, generating nucleic acids or proteins that possess viral attributes, triggering the body’s innate immune system. Secondly, they can activate the NF-κB signaling pathway and regulate pathways connected to immune responses [43]. Thirdly, they can be identified as either pathogen-associated molecular patterns (PAMPs) or danger-associated molecular patterns (DAMPs) by the immune system’s pattern recognition receptors (PRRs), prompting an immune reaction against tumors [44,45].

These viral elements can be found on the cell surface and as virus-like particles. Moreover, they can operate as targets for CD8 cells, innate immune system stimulants, and antiviral gene activity catalysts. This constellation of features renders HERVs an appealing focal point for cancer immunotherapy [46,47,48]. Consequently, researchers are exploring HERVs due to their potential to cause disease and their potential in therapeutic applications for viral infections and cancer.

This review aims to provide an overview of the current understanding of how HERV-K (HML-2) affects cancer and outlines the potential avenues for therapeutic interventions.

## 2. The Disease-Inducing Potential of HERV-K (HML-2)

HERV-K, specifically the subtype HML-2, has been implicated in both normal development and potential disturbances [49] shaping the cellular landscape; thus, HERV-K (HML-2) contributes to the development of various tissues and organs [50]. However, disturbances in HERV-K (HML-2) expression have been associated with potential implications and disorders. Aberrant activation or deregulation of HERV-K (HML-2) elements has been observed in different pathological conditions. For instance, in the context of cancer, HERV-K (HML-2) is reactivated in various cancer types, including breast, ovarian, and prostate. Its expression in cancer cells has been linked to promoting cell proliferation, invasiveness, and evasion of immune responses, potentially contributing to tumor progression [51].

HERV-K (HML-2) has also been associated with autoimmune and inflammatory diseases. Studies have shown that HERV-K (HML-2) transcripts and proteins can activate immune responses, producing inflammatory molecules. This activation may contribute to the development and progression of diseases such as multiple sclerosis (MS), systemic lupus erythematosus, and rheumatoid arthritis [47,48]. In addition, HERV-K (HML-2) has been associated with neurodegenerative diseases. Increased expression of HERV-K (HML-2) elements has been observed in the brains of individuals with diseases such as multiple sclerosis, amyotrophic lateral sclerosis, and schizophrenia. HERV-K (HML-2) may contribute to neuroinflammation and neuronal dysfunction via mechanisms that remain to be investigated [49]. 

Thus, HERV-K (HML-2) proviruses can be classified into two sub-types based on the presence or absence of a specific deletion. Type I proviruses express a protein called Np9, while type II proviruses express the Rec protein, which plays a role in RNA transport [41,52]. HERV-K Rec can induce viral restriction pathways in early embryonic cells. Polymorphic HERV-K (HML-2) loci with different structures have been identified, and their presence may explain why HERV-K (HML-2) can cause disease in certain individuals [53,54]. Currently, these loci are gaining attention as potential contributors to complex diseases. There is significant evidence of upregulation of HERV-K (HML-2 subtype)-derived messenger RNA (mRNA) and protein in different types of solid and liquid tumors [11]. The presence of endogenous retroviruses and the expression of proteins encoded by HERVs in disease states suggest the potential exploration of antiretroviral therapy for managing these conditions. Although direct inhibition of HERVs using HIV-1 RT inhibitors has been reported, until now, there have been no definitive positive clinical trial outcomes with these inhibitors in non-HIV applications. Several ongoing and completed clinical studies are utilizing various combination antiviral products, including RT inhibitors, to treat conditions such as cancer, bone loss, primary biliary cholangitis, Aicardi–Goutières syndrome, psoriasis, multiple sclerosis, and ALS, with some studies focusing on targeting HERV RTs. However, it remains uncertain if the engagement of HERV RT targets can be effectively achieved with these HIV drugs, highlighting the need for more potent and selective HERV RT inhibitors to explore potential therapeutic hypotheses [55,56]. 

### 2.1. Interaction between HERV and the Innate Immune System

The interaction between HERV and the innate immune system is crucial in maintaining immune homeostasis and preventing viral infections (Table 1). The innate immune system employs pattern recognition receptors (PRRs) to detect pathogen-associated molecular patterns (PAMPs), which include viral components [57]. Among the PRRs, two prominent families specialize in recognizing nucleic acids: the endosomal PRRs, including toll-like receptors (TLRs), and cytosolic PRRs, including RIG-I-like receptors (RLRs). Activation of TLRs triggers downstream signaling pathways, leading to the production of proinflammatory cytokines and type I interferons (IFNs) [58]. Similarly, the activation of retinoic acid-inducible gene I (RIG-I)-like receptors (RLRs) induces the production of type I IFNs and antiviral responses. It has been observed that specific PRRs, such as TLR-3 and RIG-I, can recognize HERV-derived double-stranded RNA (dsRNA), which activates innate immune responses and induces the production of type I interferons. Type I interferons are crucial in the innate immune response to HERVs. They are produced in response to recognizing HERV-derived single-stranded RNA (ssRNA) or dsRNA by PRRs [59,60,61]. These interferons inhibit HERV replication and expression, thereby limiting their activity. Additionally, type I interferons are essential in priming and activating the adaptive immune response, which further aids in controlling viral infections [52,53]. HERV-K (HML-2) has also been associated with autoimmune diseases. 

Dysregulation of the innate immune response to HERVs can significantly affect autoimmune diseases. If the innate immune system fails to control HERV expression and production of inflammatory cytokines, it can lead to chronic inflammation, tissue damage, and breakdown of self-tolerance. Some HERV env proteins contain an immunosuppressive domain (ISD) that can modulate immune responses and potentially contribute to immune tolerance, leading to the development of autoimmunity [62]. The human immune system is not tolerant to HERV-K, and similar findings have been observed in mouse models, where failures of adaptive immunity result in lethal HERV reactivation. These observations suggest that the immune response to HERV-K is unique and may not adhere to the typical tolerance seen with other self-antigens. The innate immune system is the first line of defense against pathogens, including viruses, and plays a crucial role in recognizing and responding to HERVs. The innate immune system is evolutionarily conserved and provides immediate, nonspecific immune responses. Nonetheless, it can reactivate under certain conditions, including viral infections, inflammation, and cancer. While HERVs have immunosuppressive domains, they also contribute to the development of the immune system by providing cis-regulatory elements for gene networks. They are implicated in shaping the IFN-γ network, thereby affecting the immune response. The balance between immune suppression and activation by HERVs is complex and can influence autoimmunity, malignancy, and other disease outcomes [63,64].

One interesting aspect of HERV-K-specific immune responses is the potential bystander effect. The bystander effect of HERV-K-specific immune responses refers to the possibility that the immune system may target the cells expressing HERV-K proteins and damage the surrounding healthy cells or tissues that do not express HERV-K. This could result in tissue injury, inflammation, or autoimmune reactions. For example, some studies have suggested that HERV-K expression in tumor cells may trigger an immune response that also affects the normal cells in the tumor microenvironment [65]. Similarly, HERV-K expression in neurons may induce a neuroinflammatory response that harms the neighboring glial cells [65,66,67]. The mechanisms and consequences of the bystander effect of HERV-K-specific immune responses are not fully understood. They may vary depending on the type and location of the tissue, the level and duration of HERV-K expression, and the nature and specificity of the immune response. However, some possible factors contributing to this phenomenon are as follows: (1) the fusogenic property of the HERV-K envelope protein, which allows it to mediate cell–cell fusion and create syncytia, could facilitate the spread of viral antigens or infection to adjacent cells and increase their susceptibility to immune attack; (2) the cross-reactivity of HERV-K-specific antibodies or T cells with other self-antigens that share structural or functional similarities with HERV-K proteins could lead to a loss of immune tolerance and autoimmunity; (3) the modulation of cytokine production or signaling by HERV-K proteins could affect the inflammatory response and the balance between pro-inflammatory and anti-inflammatory mediators [66,68].

The cross-reactivity of HERV-K-specific antibodies or T cells with other self-antigens is a significant point to consider. Notably, a recent study that uses bamQuery has provided valuable insights into the immunopeptidome, emphasizing the importance of quantifying RNA expression of tumor-specific antigens, including neoantigens, in both malignant and benign tissues [69]. The study shows that MHC-I-associated peptides, which can include antigens derived from non-coding genomic regions, may generate tumor-specific antigens. Moreover, the study’s findings indicate that these tumor-specific antigens can originate from multiple discrete genomic regions and are often abundantly expressed in normal tissues.

These observations are particularly relevant when considering HERV-K elements, as they constitute a significant portion of the human genome. While HERV-K elements have been implicated in various diseases, including cancer, the results produced using bamQuery suggest that the transcriptional activity of HERV-K elements may not be confined solely to pathological conditions. Instead, they may be actively expressed in normal tissues, contributing to the pool of potential antigens.

This finding underscores the complexity of HERV-K elements and their regulation, as well as the potential implications for immune responses. The presence of HERV-K-specific antigens in normal tissues raises questions about immune tolerance and the potential for cross-reactivity with HERV-K-related immune responses. As we explore the multifaceted roles of HERV-K elements in health and disease, understanding their transcriptional profile in both pathological and non-pathological contexts becomes essential.

The bystander effect of HERV-K-specific immune responses is a complex and intriguing topic that requires further investigation. It may have both beneficial and detrimental effects on human health, depending on the context and outcome of the immune response [70]. On one hand, it may enhance antitumor immunity and eliminate cancer cells expressing HERV-K. On the other hand, it may cause collateral damage to normal tissues and contribute to chronic inflammation or autoimmunity [71].

**Table 1:** Interaction between endogenous retroviruses (ERVs) and the innate immune system.

Aspect	Description	Example
Pattern Recognition Receptors (PRRs)	Innate immune system receptors detect pathogen-associated molecular patterns (PAMPs), including viral components. Among them, two prominent families specialize in the recognition of nucleic acids: endosomal PRRs (the TLR family) and cytosolic PRRs (including RIG-I-like receptors (RLRs). The toll-like receptors’ (TLRs) activation triggers downstream signaling pathways, producing proinflammatory cytokines and type I interferons (IFNs). The activation of retinoic acid-inducible gene I (RIG-I)-like receptors (RLRs) induces type I IFN production and antiviral responses.	TLR-3 and RIG-I are PRRs recognizing HERV-derived dsRNA, activating innate immune responses, and inducing type I interferon production [63].
Interferon Response	The innate immune recognition of ERVs results in the production of type I interferons (IFNs). Type I IFNs inhibit ERV replication and expression and play a crucial role in priming and activating the adaptive immune response.	HERV-derived ssRNA or dsRNA can trigger the production of type I IFNs, which enhance the immune response and limit HERV replication [63].
Epigenetic Regulation	Activation of innate immune signaling pathways induces changes in chromatin structure and DNA methylation, leading to transcriptional repression of ERVs and limitation of their activity.	Histone H3 trimethylation (H3K9me3) and DNA methylation contribute to the epigenetic silencing of HERVs and their control in differentiated cells [63].
ERV Suppression and Autoimmunity	Dysregulation of innate immune response to ERVs can contribute to autoimmune diseases. Failure to control ERV expression and production of inflammatory cytokines can lead to chronic inflammation, tissue damage, and breakdown of self-tolerance.	The transmembrane subunit of the HERV envelope protein contains an immunosuppressive domain (ISD) that can modulate immune responses and contribute to immune tolerance [63].

Moreover, we can depend on the suppression mechanisms of HERVs, which are multi-layered defenses aimed at silencing or limiting their expression, as outlined in Table 2. It is important to point out that these mechanisms may exhibit variations across different species, and our comprehension of these processes continues to evolve. Despite their general efficacy in suppressing most HERVs, specific HERV sequences have been co-opted for beneficial purposes during evolution, serving as regulatory elements for the host genome [72]. Understanding these intricate suppression mechanisms is crucial for unraveling the complex interplay between HERVs and the host immune system, shedding light on potential implications for health and disease.

HERVs contribute to autoimmunity through various mechanisms, including the production of superantigens, molecular mimicry, and DNA hypomethylation. Certain HERVs can produce superantigens that stimulate CD4 T lymphocytes, triggering an immune response associated with autoimmunity [73]. Molecular mimicry occurs when HERVs generate cross-reactive antibodies, leading to autoimmune responses. The “viral mimicry” concept has been utilized in therapy, such as the AZA-induced response [74]. This response is triggered by detecting demethylated HERVs, specifically double-stranded RNA (dsRNA), through TLR-3 in the cytosol [45]. Consequently, the production of type I interferon (IFN) is induced, leading to the apoptosis of tumor cells. However, the outcomes of this process can sometimes be controversial. Therapies targeting HERV-K-associated tumors primarily focus on suppressing invasive autoimmune responses or tumor growth by targeting HERV-K gag or Env proteins and RT.

It is theorized that beyond the env protein, additional viral proteins stemming from HERV-K (HML-2), including Rec and Np9, could potentially function as oncoproteins or initiate the production of autoantibodies within the host, thereby playing a role in the progression of diseases. Notably, the env protein has been linked to cell fusion within melanoma, culminating in tumorigenesis. Simultaneously, it is implicated in eliciting immune reactions and fostering the advancement of cancer, along with metastasis, particularly in breast cancer [45,75]. The Rec and Np9 proteins have shown interactions with disease-related proteins and potential oncogenic properties by modulating gene expression and supporting tumor progression [76]. Interestingly, HERV-K Rec not only triggers viral restriction pathways in early embryonic cells [50] but also prevents melanoma from progressing to an invasive stage [77]. Furthermore, the presence of HERVH-driven genes has been associated with improved survival in lung cancer patients, known as oncoexaptation [78]. 

LTRs bordering HERV-K (HML-2) harbor regulatory elements that can impact the expression of host genes. These LTRs exhibit transcription initiation capabilities more frequently than regular promoters, often resulting in reciprocal up- and downregulation of human genes. The effects of LTRs can be twofold, with the ability to drive the expression of tumor suppressor genes that are commonly silenced in tumors while also promoting tumorigenesis through promoter activation and gene expression alterations [79]. Furthermore, LTRs within HERV-K (HML-2) have been associated with autoimmune disorders, schizophrenia, and chromosomal rearrangements linked to overexpression of oncogenes. Thus, the regulatory functions of LTRs contribute significantly to the potential involvement of HERV-K (HML-2) in various diseases [80].

In addition, it is crucial to distinguish between active HERV transcription and passive HERV transcription. Passive HERV transcription refers to instances where HERVs are present in a zone of active transcription and are co-transcribed with other genes [81,82,83]. This distinction becomes particularly relevant in diseases like multiple sclerosis (MS), where the role of HERVs is debated. Some studies suggest that HERVs may serve as passive markers of active transcription zones, while others propose that HERVs could act as triggers of inflammation in MS. This ongoing debate highlights the need for further research to elucidate the precise mechanisms by which HERVs may contribute to the pathogenesis of diseases such as MS [82,84,85].

Polymorphic integrations of HERV-K (HML-2) within the human genome further contribute to disease susceptibility by influencing viral protein production and regulation of host genes. Specific polymorphic HERV-K (HML-2) loci have been identified with neurologic and immunologic diseases, such as Sjogren’s syndrome, multiple sclerosis, systemic lupus erythematosus, and rheumatoid arthritis [86]. However, the relationship between polymorphic integrations and diseases needs consistent observation across different studies. While autoimmune disorders have received more attention in the context of polymorphic integrations, investigations into cancer-related polymorphic integrations remain limited. 

The role of HERV-K in immune-related diseases is a complex and evolving area of research. It is important to distinguish between aberrant immunity to HERVs and aberrant expression of HERVs to understand their potential causal roles in different diseases. Aberrant immunity to HERVs refers to the immune system’s response to the presence of HERV antigens. As retroviral elements, HERVs can produce various proteins, including envelope and gag proteins, which can be recognized as foreign by the immune system. In some cases, individuals with certain diseases may have an abnormal or dysregulated immune response to HERV antigens. This immune response can lead to inflammation, autoimmunity, or other immune-related problems. However, it is crucial to note that aberrant immunity to HERVs may not be the sole cause of the disease. It could be just one contributing factor among various genetic, environmental, and epigenetic factors.

On the other hand, aberrant expression of HERVs refers to the abnormal activation or upregulation of HERV elements in specific cells or tissues. In certain diseases, such as cancer, autoimmune disorders, and neurological conditions, there is evidence of increased expression of HERVs. The abnormal expression of HERVs can result from various triggers, such as infections, inflammation, or epigenetic changes. This dysregulated expression of HERVs can lead to the production of viral proteins and RNA, which can activate immune responses and contribute to the pathogenesis of the disease.

In some cases, aberrant immunity to HERVs and aberrant expression of HERVs may be interconnected. The immune response to the abnormal expression of HERVs can, in turn, contribute to immune dysregulation and inflammation. This creates a complex feedback loop where HERVs and the immune system interact to influence disease development and progression. Therefore, the same immunity does not cause the same disease despite the presence of HERVs, which highlights the multifactorial nature of these diseases. While HERVs may be involved in various immune-related disorders, their impact can vary depending on other genetic and environmental factors, as well as the specific context of each disease. The interplay between HERVs and the immune system is likely influenced by the overall genetic background of the individual, the state of the immune system, and the presence of other pathogens or triggers. Additionally, the location and timing of HERV expression within the body could also contribute to the diversity of disease manifestations. This hypothesis remains our speculation on a feed-forward mechanism of HERV-K reactivation which may well be relevant, and it should be noted that most inflammatory conditions do not leave a lasting HERV-K reactivated state.

HERVs play a complex role in immune-related diseases, and the interplay between HERV immunity and expression is likely a contributing factor in the pathogenesis of these diseases. However, it is essential to recognize that HERVs are just one piece of the puzzle, and their impact on disease development and progression is influenced by various other factors. 

### 2.2. HERV-K (HML-2) and Its Link to Viral Infections

This section explores the role of HERV-K (HML-2) reactivation in viral infections to establish a cohesive understanding of its impact on disease development (Table 3). One of the most prominent examples of HERV-K (HML-2) reactivation is observed in HIV-1 infection [87]. HIV-1 infection activates the HERV-K (HML-2) loci, leading to the expression of HERV-K proteins [88]. The exact mechanism of HIV’s direct molecular trigger of HERV-L (HML-2) reactivation is not fully understood, but some studies have shed light on the potential interactions involved.

During HIV infection, the virus produces its regulatory proteins, such as Tat. Tat plays a crucial role in HIV replication by enhancing the transcription of viral genes [89]. HIV infection induces chronic inflammation, leading to the release of pro-inflammatory cytokines and chemokines. These inflammatory mediators can influence the expression of cellular genes, including those of HERVs. Some HERV elements, like LTRs, contain regulatory regions that respond to inflammatory signals, further promoting their transcription. Moreover, HIV infection can cause alterations in the host cell’s epigenetic landscape. Lastly, HIV’s interaction with host factors, such as cellular transcription factors and chromatin remodeling complexes, might indirectly impact the transcriptional control of HERV-L (HML-2) elements. Interestingly, Li et al. investigated HERV-K (HML-2) activation differences between HIV-1 subtype B and non-subtype B infections (CRF01_AE and CRF07_BC). It was found that subtype B infection upregulated HERV-K (HML-2) gag expression, while non-subtype B infections upregulated HERV-K (HML-2) pol expression. The genetic sequences of HIV-1 subtypes were compared, and differences in gene homology were suggested as a reason for the variations in HERV-K (HML-2) activation. The study also explored the role of HIV-1 Tat protein in HERV-K (HML-2) activation, indicating that Tat from different subtypes may have varying effects. The activation of HERV-K (HML-2) by HIV-1 is a complex process, and further research is needed to understand the underlying mechanisms [90]. The extent of HERV reactivation can vary between individuals and may depend on the viral load, immune response, and genetic factors. The human genome contains various solo LTRs and HERV sequences that are remnants of ancient viral infections. These viral parasites have successfully evaded elimination by the host. While they may not directly impact host fitness, they serve as valuable sources of regulatory elements and genes that can benefit the host organism. In the case of HIV-1 and other exogenous viruses, the infection activates the HERV-K (HML-2) loci. However, the specific impact of HIV-1 infection on regulatory ERV elements and their influence on gene expression has yet to be fully understood [88,91]. 

A recent RNA-seq study found that HIV-1 infection in primary CD4+ T cells activates multiple solo-LTRs from the ERV9 lineage, including LTR12C repeats near antiviral genes. These LTR12C elements are enriched in HIV-1-induced ERVs with transcription start sites. Two HIV-1-responsive LTR12C repeats were identified, acting as promoters for guanylate-binding proteins 2 and 5 (GBP2 and GBP5). These specific LTR12C repeats, unique to greater apes, are associated with heightened cytokine responsiveness in antiviral genes, as seen in comparative studies across primate species [88,91]. The study also shows that GBP2 and GBP5 decrease the infectiousness of HERV-K (HML-2) pseudoparticles, suggesting that these LTR-induced host factors have already been shown to be useful against other viral pathogens [92]. These findings illustrate how human cells utilize retroviral remnants to enhance innate immune responses against contemporary viruses. Additionally, another study linked the viral protein Tat, crucial for HIV replication and pathogenesis, to the induction of HERV-K (HML-2) expression. Tat directs cellular transcription machinery to HERV-K (HML-2) long terminal repeats (LTRs), promoting its transcriptional activation. The activation of HERV-K (HML-2) in HIV-infected individuals has spurred interest in targeting it therapeutically, with various approaches explored, including antiretroviral drugs and antibodies [93,94]. 

In COVID-19, interferon (IFN) plays a crucial role in the innate immune signaling pathway and host antiviral immunity. The study discussed the activation of human endogenous retroviruses (HERVs), specifically HERV-K (HML-2), with interferon secretion. High expression of HERVs, including HERV-K, has been observed in COVID-19 patients. A study by Guo et al. divided COVID-19 patients into moderate and severe groups and investigated the activation of HERV-K (HML-2) and the expression of interferon-related genes. They observed significant upregulation of HERV-K (HML-2) genes in COVID-19 patients and its association with increased interferon levels, providing insights into the mechanism of interferon production in COVID-19. The study also explored the expression of interferon-related genes in a monkey kidney cell line and suggested using a cell line that can adapt to viral infection and secrete interferon for more effective vaccine production [95]. The findings contribute to understanding the activation of HERV-K (HML-2) in COVID-19 and its potential implications for immune responses and disease progression. In severe COVID-19 cases, there is an uncontrolled release of pro-inflammatory mediators, leading to inflammation and tissue damage. Immune cells, such as neutrophils and monocytes, contribute to inflammation and tissue damage in COVID-19. Temerozo et al. performed a virome analysis of critically ill COVID-19 patients and identified the activation of endogenous retroelements, including HERV-K. This analysis showed that not only COVID-19 antivirals and anti-inflammatory medications suppressed this expression. HERV-K expression increased in the lower respiratory tract and plasma of severe COVID-19 patients, particularly in those who died, also increased HERV-K expression and is associated with upregulation of pro-inflammatory markers, monocyte activation, and coagulopathy. SARS-CoV-2 experimental infection of human primary monocytes caused the expression of HERV-K to increase; COVID-19 antivirals and anti-inflammatory medications suppressed this expression. These findings link HERV-K to the physiopathology of COVID-19 patients who are critically unwell [96]. 

A high-throughput analysis of specific HERV loci in PBMCs was conducted on healthy controls, convalescent individuals, and those retesting positive after recovering from SARS-CoV-2 infection. Differentially expressed HERV loci (deHERV) were identified in individuals exposed to SARS-CoV-2 infection, regardless of the disease’s clinical form. Distinct deHERV loci were found in convalescent individuals and those retesting positive compared to healthy controls. These HERV loci encompassed various HERV groups, including all three classes. This study underscores the connection between HERV expression in PBMCs and the clinical manifestation and prognosis of COVID-19, shedding light on the interplay between HERVs and cellular immunity. It also provides specific transcriptional patterns that may influence COVID-19’s clinical presentation and course of action [97]. 

The relationship between human T-cell leukemia virus type 1 (HTLV-1) and HERV-K (HML-2) is of interest in understanding HTLV-1-associated diseases. While HTLV-1 typically remains asymptomatic in most infected individuals, some develop severe conditions like adult T-cell leukemia/lymphoma (ALT) and HTLV-1 HAM/TSP. The mechanisms underlying these diseases remain unclear, but HTLV-1 infection leads to inflammation and neurological symptoms by accumulating infected T cells in the CNS. Damage to uninfected cells may occur due to bystander effects or cross-reactivity of CD8+ T cells with self-antigens in neurons. HERV-K (HML-2) is implicated in this context, given the similarities between HAM/TSP and multiple sclerosis, where HERV expression is associated with disease. Recent studies have shown that the HTLV-1-Tax protein induces the transcription of HERVs [98]. Screening of HTLV-1-infected individuals for T-cell responses to HERV-derived peptides, specifically focusing on the HERV-K (HML-2) lineage, has provided indirect evidence for the in vivo expression of HERV antigens. The detection of HERV-specific T-cell responses in HTLV-1-infected individuals raises questions about their potential contribution to the pathogenesis of HAM/TSP [99]. It is hypothesized that HTLV-1 infection induces the expression of HERV antigens, leading to the activation of HERV-specific T-cell responses that could potentially target neurons and other tissues expressing low levels of HERV antigens. Further investigations are necessary to determine the extent of HERV involvement in HTLV-1-associated diseases and their contribution to the observed pathologies.

Interestingly, the expression of HERV-K (HML-2) in hepatitis C virus (HCV) infection has been shown to have implications for liver damage and treatment. The results indicate that factors beyond inflammatory pathways influence HERV-K (HML-2) expression and may be associated with impaired liver function. For instance, increased HERV-K (HML-2) transcript levels are significantly correlated with elevated non-invasive blood markers like ASAT, ALAT, and albumin, which are indicators of liver damage and worsened disease prognosis. They suggested the potential inclusion of reverse transcriptase inhibitors, such as Raltegravir, in HCV antiviral therapy to improve treatment success. These inhibitors have proven effective in treating retroviral diseases like HIV and have shown the ability to inhibit human endogenous retroviruses [100]. Similar associations between HERV activation and disease development have been observed in conditions such as multiple sclerosis (MS), triggered by Epstein–Barr virus (EBV) infection. In EBV-immortalized lymphoblastoid B cell lines (LCL) from MS-affected individuals, increased expression of HERV-K loci and other genes associated with relapses was observed, indicating a potential role of HERVs in MS pathogenesis [101]. These findings underscore the importance of understanding the role of HERVs in viral infections and their potential as biomarkers or therapeutic targets in related diseases (Table 3). Apart from direct molecular triggers, inflammation, and cellular senescence can also induce HERV-K (HML-2) reactivation. Cytokine-induced senescence is a common feature in many viral infections and could be of particular importance in viral-associated diseases like HCV [66]. Studies have shown that HERV-K (HML-2) is maintained after HCV viral clearance and is associated with the fibrosis score, suggesting its potential role in liver damage and disease progression.

Overall, the activation of endogenous retroviruses, such as HERV-K (HML-2), in the context of viral infections like HIV, HTLV-1, and COVID-19, highlights the intricate relationship between viral pathogens and the human genome. While these viral remnants have the potential to modulate immune responses and contribute to disease pathogenesis, they also represent a fascinating evolutionary record of ancient viral infections. They may offer opportunities for the development of novel therapeutic strategies.

### 2.3. Exploring the Expression of HML-2 in Tumors

Earlier research has indicated that human endogenous retrovirus (HERV) can induce the proliferation of tumor cells and evade apoptosis, representing a significant contributing factor to tumor advancement. HML-2 expression has been detected in various types of tumors, and its presence has been associated with prognostic features and unfavorable outcomes in these cancers (Table 4). HERV-K (HML-2) colonized the human germ line and is closely related to the mouse mammary tumor virus (MMTV) that causes breast cancer in mice [102]. In breast cancer, the overexpression of HML-2 is linked to aggressive subtypes and the spread of cancer cells to lymph nodes. Similarly, HML-2 is upregulated in basal-like breast cancers, especially in triple-negative breast cancer cases [103,104]. Notably, recent research has identified shared CD8+ T-cell epitopes derived from cancer-associated HERVs, including those from the HML-2 family, in solid tumors [105]. These epitopes have shown immunogenicity and the ability to induce high-avidity CD8+ T-cell responses. This discovery holds significant promise for the development of cancer vaccines or T-cell-based immunotherapies, particularly in tumors with high expression of HERVs from the HML-2 family, such as triple-negative breast cancer cases in prostate cancer, HML-2 expression is higher than in benign tissues, and it correlates with tumor development and metastasis [96,106]. Melanoma also exhibits HML-2 expression, associated with tumor progression and decreased survival [107]. Regarding HERVs in leukemia, recent research demonstrated the expression of HML-2-derived HERVs in leukemic stem cells. It highlighted the presence of HERV-specific CD8+ T cells in the bone marrow of leukemia patients. Further contributing to the development of a HERV signature that can be used to characterize leukemic stem cells [33]. In lung cancer, HML-2 can be detected in blood samples of patients, although its levels do not correlate with the stage of the disease [108]. Additionally, hepatocellular carcinoma and colorectal cancer display increased HML-2 expression, linked to poorer overall survival. Furthermore, HML-2 is observed in leukemia and lymphoma, suggesting its potential involvement in hematological malignancies.

HERV-K sequences are highly expressed in teratocarcinoma cells and have been associated with leukemia and germ cell tumors [109,110]. Recent advancements in PCR-based target enrichment sequencing protocols have provided valuable information about the role of HERV-K (HML-2) in different cancers. The detection of HML-2 mRNA in the blood shows promise as a particular disease marker for multiple cancers [111]. For instance, in breast cancer, HML-2 env mRNA levels were significantly elevated in patients’ blood, and chemotherapy was found to reduce its expression. Notably, Wang-Johanning et al. discovered that serum HML-2 mRNA was upregulated in women with early-stage ductal carcinoma in situ (DCIS), indicating its potential as an early marker for metastatic risk. Prostate-specific antigen (PSA) is currently used for prostate cancer screening, but alternative markers are sought due to its limitations [112]. Wallace et al. explored the detection of HML-2 in peripheral blood mononuclear cells (PBMCs) as a combined test. They observed significant upregulation of HML-2 gag mRNA in prostate cancer cases compared to controls, with its levels associated with higher odds of diagnosis. Interestingly, HML-2 gag exhibited better predictive ability in older men compared to PSA [113].

Regarding the functional role of HML-2 in tumor progression, its expression has been associated with disease advancement in various cancer types, suggesting a functional role rather than a mere consequence of carcinogenesis. Breast cancer, pancreatic cancer, leukemia, and teratocarcinoma cells have been discovered to enhance cell proliferation in response to HML-2 proteins such as env and Np9. Inhibiting HML-2 env in breast cancer reduced cell invasion and migration by affecting the Ras/Raf/MEK/ERK signaling cascade. Similarly, cell migration in assays for wound healing was impacted by the modification of Np9 expression in teratocarcinoma cells [37,114]. Furthermore, HML-2 Env, which contains a potential immunosuppressive domain, may possess immunomodulatory capacity as a non-self-epitope. Treatment with HML-2 viral particles or a recombinant peptide derived from the transmembrane subunit inhibited immune cell proliferation and altered cytokine secretion, including an increase in immunosuppressive interleukin-10 (IL-10). This suggests that HML-2 env might interact with tumor-infiltrating immune cells, contributing to the development of an immunosuppressive tumor microenvironment. The specific mechanisms of interaction and signaling pathways involved require further investigation [115,116].

Additionally, it has been discovered that the expression of HML-2 is a distinct marker for embryonic stem cells (ESCs) and induced pluripotent stem cells (iPSCs), being quickly muted following differentiation. Due to this property, HML-2 has been investigated as a marker for cancer stem cells (CSCs) or tumor-initiating cells. When melanoma cells were grown in stem-cell media, HML-2 transcription increased, correlated with improved stemness traits and the proliferation of putative CSC populations. HML-2 suppression stopped the CD133+ CSC population from growing [117]. Furthermore, stem/progenitor cell counts in samples from leukemia patients were linked with Np9 protein levels [116,118]. It is important to acknowledge that although HML-2 proteins have the potential to play functional roles in tumor progression, further research is required to fully comprehend the underlying molecular mechanisms and their implications for cancer development and treatment. HERV-K proteins are characterized as neoantigens, which are tumor-specific antigens expressed in various cancer types. They can trigger both innate and adaptive immune responses. Innate immunity activates B and T cells to produce antibodies and cytotoxic T-cell responses [48]. Studies have shown that antibodies targeting HERV-K can inhibit cancer growth in vitro and in animal models. HERV-K expression has been studied as a biomarker for cancer screening and a target for immunotherapy. Several studies have demonstrated immune responses, including the production of anti-HERV-K antibodies and HERV-K-specific CD8 T-cell responses, against HERV-K in these types of cancer. In melanoma, cytotoxic CD8 T-cell responses against HERV-K have been observed, and the prevalence of anti-HERV-K antibodies has been found to differ significantly between melanoma patients and healthy individuals [119]. In prostate cancer, HERV-K expression has been associated with disease progression and poorer survival outcomes [120]. Similarly, in breast cancer, HERV-K expression has been correlated with a negative prognosis and has been suggested as a potential prognostic biomarker. Additionally, HERV-K antigens have been investigated as potential targets for cancer immunotherapy, including dendritic vaccines, CAR-T cells, and recombinant vaccines [121]. Unlike conventional tumor antigens, HERV-K (HML-2) exhibits a distinct complexity, composed of protein and nucleotide components, making it a versatile antigen. This intriguing combination endows HERV-K (HML-2) with diverse characteristics, including cell surface presentation, viral particle formation, CD8 T-cell targetability, and the ability to stimulate innate immune responses. These features have sparked interest in exploring the potential of HERV-K (HML-2) as a novel immunotherapeutic target for cancer treatment.

**Table 4:** Main ideas of HERV-K (HML-2) as a target in cancer.

Type of Cancer	Key Ideas of HERV-K (HML-2) as a Biomarker and/or a Target in Cancer
Breast Cancer	-HERV-K (HML-2) expression correlates with disease progression, lymph node metastasis, and reduced overall survival. -HERV-K env protein shows promise as a therapeutic target in immune-mediated therapies. Anti-HERV-K monoclonal antibodies (mAbs) and HERV-K env-specific CAR-T cells have effectively reduced tumor growth [60,122].
Hepatocellular Carcinoma	-HERV-K (HML-2) expression is an independent prognostic indicator of overall survival. -The HERV-K env protein is associated with cirrhosis, tumor differentiation, and staging [60].
Hodgkin’s Lymphoma	-Evidence suggests HERV-K expression in patients with Hodgkin’s lymphoma, with a significant drop in titer levels after appropriate treatment [60].
Melanoma	-HERV-K (HML-2) expression has been observed in melanoma cells, and HERV-K-specific CD8+ T-cell responses have shown specificity to HERV-epitope-presenting tumor cells [119]. -The HERV-K env protein is a potential therapeutic target.
Prostate cancer	-HERVs could potentially serve as diagnostic or prognostic biomarkers for prostate cancer due to antibody response [61,123].
Germ Cell Tumors	-The expression of HML-2 and its co-correlation with other genes, such as proline dehydrogenase (PRODH), suggests its involvement in tumorigenesis, particularly in germ cell tumors [124]. The epigenetic regulation of HML-2 expression and its potential role as a cis-regulatory element highlights the possibility of targeting HML-2 to regulate tumor-specific gene expression [62,125].
Ovarian Cancer	-Detection of antibody response. The immune system can recognize the abnormal expression or activation of HERVs in ovarian cancer cells through TLR3 and MAVS, leading to the production of type I interferon (IFN) and triggering apoptosis [63].
Pancreatic Cancer	-HML-2 env has been shown to promote proliferation [64,126].
Hepatocellular Cancer	-HERV-K (HML-2) expression in colorectal cancer is correlated with clinical parameters such as cirrhosis, tumor differentiation, and TNM stage. Additionally, high expression of HERV-K (HML-2) is associated with poorer overall survival in colorectal cancer patients, indicating its potential as a prognostic biomarker for this type of cancer [65].
Lung Cancer	-The activation of B cell and antibody responses against these HERV antigens may play a significant role in anti-tumor immunity and the response to immunotherapy in lung cancer patients–HERV has a potential biomarker [66,67,127].
Colon Cancer	-HERV-K (HML-2) expression has been implicated in colon cancer progression. Studies have shown that increased HERV-K (HML-2) expression correlates with disease progression, lymph node metastasis, and reduced overall survival in patients with colon cancer. -Targeting HERV-K (HML-2) proteins, such as the env protein, may hold promise as a therapeutic strategy in colon cancer [68].
Colorectal Cancer	Differentially expressed HERV-K (HML-2) loci in colorectal cancer were identified, with a concentration in immune response signaling pathways, indicating the potential impact of HERV-K on the tumor-associated immune response. These findings suggest that HERV-K could serve as a screening tumor marker and a target for tumor immunotherapy in colorectal cancer [69].
Acute Myeloid leukemia	A study utilizing whole-genome sequencing and read mapping identified a statistical correlation between AML and 101 HERV-K (HML-2) transposable element insertion polymorphisms (TIPs), indicating a potential relationship between HERV-K (HML-2) and AML [70].
Glioblastoma	HERV expression was associated with a cancer stem cell phenotype and poor patient outcomes. Inhibiting HERV-K (HML-2) expression with antiretroviral drugs can reduce tumor viability and pluripotency [128].
Renal Carcinoma	HERV-K (HERV-K env) was identified as a novel tumor antigen and prognostic indicator. Higher HERV-K (HML-2) env protein levels are associated with better disease-specific survival rates, suggesting potential as a prognostic marker [129].

Recent research in glioblastoma found a pathological expression of HERV-K (HML-2) in both cerebrospinal fluid and tumor tissue. This expression was associated with a cancer stem cell phenotype and poor patient outcomes. The virus was particularly active in neural progenitor-like cells, driving cellular plasticity and maintaining stemness, which are characteristics associated with tumor aggressiveness and resistance to treatment. Inhibiting HERV-K (HML-2) expression with antiretroviral drugs reduced tumor viability and pluripotency, suggesting that it could be a promising therapeutic target to tackle treatment resistance and recurrence in glioblastoma [130].

Similarly, HERV-K (HML-2) influenced tumorigenic characteristics in colorectal cancer. Knocking out the HERV-K env gene using the CRISPR-Cas9 system in colorectal cancer cells significantly reduced cell proliferation, migration, and tumor colonization. This effect was related to the downregulation of nuclear protein-1 (NUPR1), which plays a crucial role in cancer cell proliferation and migration. Inhibition of HERV-K (HML-2) expression decreased reactive oxygen species levels, further affecting cancer cell behavior [131]. In the case of ovarian cancer, the CRISPR-Cas9 knockout of the HERV-K (HML-2) env gene affected tumorigenic characteristics in different ways depending on the ovarian cancer cell line. Knocking out the gene led to cell proliferation, migration, and invasion changes by affecting the expression of RB and Cyclin B1 proteins, critical regulators of cell cycle progression and tumor growth [132].

In renal cell carcinoma, the envelope protein of HERV-K (HERV-K env) was identified as a novel tumor antigen and a prognostic indicator. Its expression was significantly elevated in clear renal cell carcinoma (ccRCC) compared to other subtypes. Patients with higher HERV-K (HML-2) env protein levels had better disease-specific survival rates, suggesting its potential as a prognostic marker in this type of cancer [133].

The findings from these studies highlight the significant role of HERV-K (HML-2) in various types of cancer, influencing critical aspects such as stem cell characteristics, tumorigenic behaviors, and patient prognosis. The reactivation of these endogenous retroviruses presents exciting new opportunities in cancer research, including potential applications in diagnostics, therapeutic targeting, and prognostic indicators. However, to harness the full potential of HERV-K as a clinical tool, further research is essential to comprehensively understand its underlying mechanisms and clinical implications in cancer development and treatment. To optimize HERV-K as a specific target for different malignancies, more in-depth characterization of HERV-K expression patterns in various cancer types is required. In developing therapeutic strategies, it is crucial to consider how HERV-K contributes to normal development and whether its targeting could disrupt essential cellular processes. This requires a thorough understanding of the functional roles of HERV-K in healthy cells and tissues. By carefully assessing its impact on normal physiology, researchers can design therapies explicitly targeting cancer cells while sparing healthy cells. To validate the effectiveness of HERV-K targeting in cancer management, it is imperative to conduct comprehensive in vitro models and robust clinical research. In vitro studies will provide valuable insights into the mechanisms of HERV-K action and its interactions with various signaling pathways, aiding in the development of targeted therapeutics. Clinical trials will be crucial to evaluate the safety, efficacy, and potential side effects of HERV-K-based therapies in real-world settings.

Moreover, HERVs hold promise not only as therapeutic targets but also as potential cancer biomarkers. Monitoring HERV-K expression levels in cancer patients could provide valuable diagnostic and prognostic information, facilitating early detection and personalized treatment strategies. HERV-K (HML-2) represents an exciting avenue in cancer research, with potential applications in diagnostics and targeted therapies. However, translating these findings into clinical practice requires extensive research and validation in diverse cancer types. By advancing our understanding of HERV-K biology and its role in cancer, we can unlock the full potential of these endogenous retroviruses as therapeutic targets and biomarkers for improved cancer management. 

## 3. Exploring Therapeutic Opportunities: Potential Drug Targets of HERV-K (HML-2) and Existing Medication

### 3.1. Potential Use of Antiviral Drugs 

HERV-K (HML-2) is a retrovirus that can encode various enzymes critical for its replication and integration into the human genome. One such enzyme is reverse transcriptase, which converts the viral RNA genome into DNA. This process is crucial for integrating HERV-K (HML-2) DNA into the host cell’s genome, making it a potential target for antiviral drugs [134]. Non-nucleoside reverse transcriptase inhibitors (NNRTIs) are antiviral drugs explicitly targeting the reverse transcriptase enzyme. By inhibiting this enzyme, NNRTIs could disrupt the reverse transcription process of HERV-K (HML-2), leading to reduced viral DNA synthesis and integration into the human genome. In addition to reverse transcriptase, HERV-K (HML-2) also encodes protease enzymes. Protease cleaves viral polyproteins into their functional components during the maturation of viral particles [135]. Inhibition of HERV-K (HML-2) protease could interfere with the proper maturation of viral particles. This may produce noninfectious HERV-K (HML-2) particles, significantly reducing their ability to spread and infect new cells. Targeting the protease enzyme provides another potential avenue for developing antiviral therapies against HERV-K (HML-2). Furthermore, HERV-K (HML-2) relies on its integrase enzyme to integrate its DNA into the human genome, a critical step in its replication cycle [136]. Integrase inhibitors are a class of antiviral drugs that specifically block the action of integrase enzymes. Using integrase inhibitors, researchers could prevent the stable integration of HERV-K (HML-2) DNA into the host cell genome. This could lead to incomplete replication of the virus and a reduced ability to spread to other cells. HERV-K (HML-2) integrase is similar to HIV integrase in its structure and function but has some differences in its sequence and specificity. HERV-K (HML-2) integrase prefers integrating into gene-rich genome regions, whereas HIV integrase has a more random distribution. HERV-K (HML-2) integrase also interacts with different cellular proteins than HIV integrase, which may affect its activity and regulation. For example, HERV-K (HML-2) integrase binds to a protein called TRIM28, which is involved in gene silencing and DNA repair. This interaction may help HERV-K (HML-2) evade host defense mechanisms and increase its integration efficiency [137]. 

Recent studies have revealed the potential of specific antiviral drugs (listed in Table 5) to inhibit the expression of HERV-K (HML-2), which opens up possibilities for treating diseases associated with HERV-K (HML-2) activation. However, further research is necessary to fully understand the underlying mechanisms and the relationship between antiviral drugs and HERV-K (HML-2) expression. Researchers have explored the crystal structure of HERV-K HML-2 RT and compared it to HIV-1 RT, revealing similarities and differences between the two enzymes. The structural differences provide insights into HERV-K RT as a potential therapeutic target, although further research is necessary to understand its role in disease causation and develop selective inhibitors [138]. Some antiretroviral drugs approved for treating HIV infection have shown effectiveness against HML-2 in vitro. In ALS patients, studies have investigated antiretroviral combination therapy, including drugs like abacavir, lamivudine, and dolutegravir. Preliminary observations suggested a decrease in HML-2 levels with this treatment. Comprehensive studies have demonstrated sustained reductions in HML-2 levels throughout treatment, and discontinuation of the therapy led to a rapid increase in HML-2 levels, indicating the direct antiviral effect of the drugs [138]. While most antiretroviral drugs are less potent against HML-2 than HIV, abacavir has shown greater efficacy against HML-2. Both lamivudine and abacavir can penetrate the blood–brain barrier, making them potential candidates for treating diseases like HIV and HML-2 associated neurological disorders [139]. Other studies have explored the effectiveness of integrase inhibitors and HIV-protease inhibitors against HML-2: one group showed that darunavir and lopinavir, two protease inhibitors, quickly docked to the protease catalytic site and decreased the activity of the HML2 protease, while the integrase inhibitor raltegravir hindered the replication of the VSV-G pseudotyped HML2 in a dose-dependent manner. This group also hypothesized that HML2 replication would be dependent on integration because the integrase inhibitor raltegravir strongly inhibited HML2 integrase. According to the research mentioned above, it appears possible that the ARVs used in HIV treatment can be employed to combat the HERVs expressed in various cancer types [136,140]. Interferon-beta, known for its antiviral activity, has also been investigated in ALS patients but did not show significant differences compared to a control group [141]. Moreover, in 2019, Rigogliuso et al. found that a human endogenous retrovirus-encoded protease, specifically HERV-K (HML-2) Protease (Pro), potentially cleaves numerous cellular proteins [135]. The researchers used a modified terminal amine isotopic labeling of substrates (TAILS) procedure to identify human cellular protein substrates of HERV-K (HML-2) Pro. They found that HERV-K (HML-2) Pro significantly processed thousands of human proteins at both acidic and neutral pH. These findings suggest that even low-level expression of HERV-K (HML-2) Pro could affect levels of a diverse array of proteins and thus have a functional impact on cell biology and possible relevance for specific diseases [49,142].

Antiretroviral drugs, initially designed for treating HIV-1 infection, have successfully suppressed HIV replication. There are 32 FDA-approved antiretroviral drugs for HIV/AIDS treatment, targeting reverse transcriptase, integrase, and proteinase. Given the similar genomic structure between HERV-K and HIV, antiretroviral drugs developed for HIV have the potential to inhibit HERV-K, Table 5. 

Overall, inhibiting HERV-K (HML-2) expression by antiviral drugs represents a potential therapeutic approach for diseases associated with HERV-K activation. Further research is necessary to understand the mechanisms of action and develop selective inhibitors. Identifying and validating effective treatments targeting HERV-K could lead to improved outcomes for patients with autoimmune disorders and HERV-K-associated tumors.

### 3.2. HERV-Based Therapies for Cancer

The potential role of HERV-K (HML-2) in cancer and autoimmunity makes it an attractive target for innovative therapeutic strategies. While HERV-based therapies are being explored, the definitive evidence of a causal association between HERVs’ presence/expression and the onset/progression of specific disorders remains to be established. A significant gap in current studies is the need for standardized methodologies and robust genomic characterization of individual HERV groups to produce consistent and reliable results [75]. HERV envs could serve as targets for both passive and active immunotherapies against cancer. In certain cancers, such as melanoma and colorectal carcinoma, HERV-K (HML-2) envs have been investigated as tumor-associated antigens (TAAs) and tumor-specific antigens (TSAgs) for stimulating immune responses against cancer cells [147,148,149,150]. Moreover, combining HERV-based immunotherapy with demethylating agents has been proposed to enhance anticancer effects. HERV-based immunotherapy with demethylating agents has been proposed to enhance anticancer effects. DNA methyltransferase (DNMT) inhibitors can activate silenced genes at low doses and cause cytotoxicity at high doses. The ability of DNMT inhibitors to reverse epimutations is the basis of their use in novel strategies for cancer therapy [151,152,153]. Recently, several papers have described favorable outcomes of increasing HERV RNA and DNA abundance by treatment of cancer cells with methyltransferase inhibitors. Analogous to an infecting agent, the ERV-derived nucleic acids are sensed in the cytoplasm, activating innate immune responses that drive the tumor cell into apoptosis. This viral mimicry induced by epigenetic drugs might offer novel therapeutic approaches to help target cancer cells that are usually difficult to treat using standard chemotherapy [154]. Regarding cancer treatment, HERV-targeted therapies offer a promising new class of treatments for cancer and biomarker strategy. The upregulation of HERV-K (HML-2) in various tumor types and its association with disease progression and poor outcomes has led researchers to explore targeting HERV-K proteins as an attractive approach for aggressive metastatic disease.

HERV-K (HML-2) has been identified as a tumor-associated antigen (TAA) expressed on various cancer cells, including breast cancer and melanoma. This makes it an attractive target for developing immunotherapeutic strategies to combat these diseases. CAR-T cell therapy is a groundbreaking approach in oncology, where T cells are genetically engineered to express chimeric antigen receptors (CARs) on their surface. These CARs enable T cells to recognize and target specific antigens in cancer cells. In the context of HERV-K (HML-2), cell-based technologies such as autologous chimeric antigen receptor T (CAR-T) cells have been designed to specifically target HERV-K envelope protein, which is overexpressed on cancer cells but not on normal cells [155,156]. These therapies have shown high efficacy in blood cancers and hold promise for future applications in targeting HERV-K proteins. CAR-T cells specifically designed to target HERV-K env have successfully suppressed breast cancer cell growth and metastasis in preclinical models, reducing tumor growth and weight in animal models. Additionally, HERV-K (HML-2)-specific CAR-T cells have demonstrated the ability to prevent tumor metastasis to other organs, indicating their potential for metastatic cancer treatment [157].

This approach demonstrates the potential of utilizing HERV-K and other transposable element-derived proteins as tumor-associated antigens (TAAs) in cell-based therapies [141,152]. Furthermore, epigenetic modifications in CAR-T cells can overcome barriers limiting their effectiveness, especially in immunosuppressive tumor microenvironments. These modifications can enhance CAR-T-cell phenotype and function, ultimately improving the overall efficacy of CAR-T-cell therapy [158].

Despite the promising results, challenges and limitations still need to be addressed in the development of CAR-T cell therapy targeting HERV-K (HML-2). Further research is required to optimize the CAR-T cell design, improve their persistence and efficacy, and overcome potential immunosuppressive factors in the tumor microenvironment. Nevertheless, the development of HERV-K (HML-2)-specific CAR-T-cell therapy represents an exciting avenue for the advancement of cancer immunotherapy and offers hope for improved treatment outcomes for patients with breast cancer, melanoma, and other solid tumors expressing HERV-K (HML-2) [155]. 

In addition to antibody-based and cell-based therapies, epigenetic therapy has shown promise in upregulating HERV-K expression and stimulating anti-tumor immune responses. Inhibiting DNA methyltransferases (DNMTs) in various cancer types has been shown to activate immune signaling pathways by sensing HERV-derived double-stranded RNA. This activation restores effector T-cell function and prevents tumor immune evasion. The upregulation of HERV-K and other TAAs through epigenetic therapy suggests combining these approaches with anti-HERV-K antibodies or cell therapies for enhanced therapeutic outcomes [67,145].

Moreover, HERV-K (HML-2) could also be exploited as an anti-cancer vaccine, particularly in tumor types where HERV-K reactivation occurs early in carcinogenesis. Preclinical studies using a modified vaccinia virus expressing HERV-K env have shown promising results in preventing tumor establishment. The development of HERV-K-based vaccines holds potential for cancer immunotherapy and could be further explored in clinical trials [121]. The application of HERV-K research extends beyond cancer and infectious diseases. For instance, clustering and classification techniques have been used to analyze HERV-K expression patterns and identify subtypes and variants associated with specific diseases [159]. This approach helps to unravel the diverse nature of HERV-K activity and its potential implications in various disorders. The modified VLV (virus-like vaccine) technology, based on adenoviral vectors encoding virus-like particles, induces T-cell immunogenicity. This is important because CD8+ T cells are critical for eliminating cancer cells. Vaccination with the modified VLV provides cross-protection against different tumor types expressing ERV-derived antigens. This indicates the potential for broad-spectrum immunotherapeutic approaches [160,161,162]. 

Comparing HERV-K to other individual antigenic classes explored for tumor immunotherapy, recent studies suggest that immunization with murine ERVs (MelARV) using VLV technology outperformed immunization studies published for neoantigens. Combining the modified VLV with an α-PD1 checkpoint inhibitor (CPI) exhibited excellent curative efficacy against established mouse colorectal tumors [161,163]. Furthermore, vaccinated mice that survived the initial tumor challenge were additionally protected against rechallenge with a different breast cancer cell line, demonstrating cross-protection against various tumor types expressing HERV-derived antigens. Recent studies have shown promising results in immunization strategies targeting murine ERVs, outperforming those published for conventional neoantigens [164,165]. This remarkable finding highlights the immense potential of HERV-K (HML-2) as an attractive and practical immunotherapeutic target for cancer treatment. By exploiting its unique antigenic properties, HERV-K (HML-2) can activate potent and specific immune responses against tumors, improving treatment outcomes. The envelope protein of HERV-K (HML-2) is particularly appealing as an immunotherapeutic target due to its cell surface expression on cancer cells. HERV-K env can stimulate innate and adaptive immunity, promoting crucial inflammatory, cytotoxic, and apoptotic reactions necessary for mounting an effective immune response against cancer [48]. 

Furthermore, advancements in gene-editing technologies like CRISPR/Cas9 have opened up possibilities for targeting HERV-K elements. In preclinical studies, CRISPR/Cas9-mediated knock-out of the HERV-K env gene has shown promising results in inhibiting its transcription and translation. This disruption of HERV-K function has implications for regulating fundamental cellular processes and could pave the way for novel therapeutic approaches in cancer treatment [166,167]. While the potential of HERV-K (HML-2)-targeted therapies is promising, it is essential to exercise caution due to the potential role of HERV-K in normal human physiology, its expression in stem cells and the placenta, and the need for a comprehensive understanding of HERV-K expression in healthy human tissues [168]. 

Finally, we would like to highlight the utilization of antiviral agents for cancer management. Although antiviral agents were initially developed to combat viral infections, they have shown intriguing potential for oncology. Emerging evidence suggests that certain viruses, including human endogenous retroviruses (HERVs), play a role in cancer development and progression [169,170]. Since viral elements are integrated into the human genome and can contribute to cellular transformation and tumorigenesis by employing antiviral agents, it is possible to disrupt the viral machinery within cancer cells, impeding their growth and survival. When strategically combined with established anticancer therapies, antiviral agents present a two-pronged attack against cancer. Conventional chemotherapy and radiotherapy primarily target rapidly dividing cancer cells, but their effectiveness can be limited by tumor heterogeneity and the development of resistance. Integrating antiviral agents into the treatment regimen adds a complementary layer of action. Antivirals may render tumor cells more susceptible to the cytotoxic effects of chemotherapy or radiation by inhibiting viral components within cancer cells, potentially overcoming resistance and enhancing the overall therapeutic response [171,172]. The combination approach of antivirals and anticancer agents can address two critical challenges in cancer treatment: immunosuppression and metastasis. Tumors often create an immunosuppressive microenvironment, which hampers the body’s natural immune response against cancer cells. By suppressing viral activity, antiviral agents can contribute to reversing immunosuppression and reactivating immune surveillance against tumors. When coupled with the direct cytotoxicity of anticancer therapies, this immunomodulatory effect could stimulate a more robust immune-mediated attack on cancer cells [173].

Therefore, the investigation of HERV-K (HML-2) as a potential drug target and the development of antivirals for viral infections and cancer treatment offer promising therapeutic opportunities that hold the potential to revolutionize the field of medicine. Table 6 illustrates the limited number of clinical trials related to HERV-K, highlighting the long journey ahead in the quest to enhance HERV-K-based therapies. Despite the challenges, continued research and exploration of HERV-K could unlock new avenues for innovative treatments and benefit patients facing various medical conditions.

## 4. Exploring HERV-K (HML-2) to Unravel Disease Mechanisms for Improved Treatment Strategies: In Vitro and In Silico Approaches

Extensive research has examined the role of HERV-K, specifically the HML-2 subtype, in cellular physiology and its relationship with disease pathogenesis. Studies that demonstrated an antiviral effect against HML-2 showed a trend for slower progression of the disease, which supports the possible involvement of this virus in the pathogenesis of diseases. Searching for antivirals that either alone or in combination with each other influence the viral cargo of HERV-K (HML-2) can be advantageous to better understand the mechanism of action of this virus and finding treatments in the case of HERV-K (HML-2) reactivation. Regarding cancer treatment, we could seek a combination of antivirals with chemotherapy. We aim to contribute to this understanding by employing a perspective approach for future research that combines in vitro studies and in silico analyses.

The detection of viral cargo in microvesicles has important implications for the diagnosis and treatment of diseases. The viral cargo delivered by microvesicles can induce prominent immunomodulation in recipient cells, which may contribute to developing diseases. Therefore, understanding the role of microvesicles in the assembly and transmission of viruses can help identify potential targets for therapeutic intervention. Exploring HERV-K (HML-2) in teratocarcinoma cell lines and microvesicles can help unravel disease mechanisms for improved treatment strategies in several ways. Firstly, by identifying the role of microvesicles in the assembly and transmission of viruses, this research can help identify potential targets for therapeutic intervention. Secondly, researchers can develop new strategies for diagnosing and treating diseases associated with these viruses by understanding how HML-2 elements are captured during the shedding of microvesicles and delivered to recipient cells. Thirdly, by studying the immunomodulatory effects of viral cargo delivered by microvesicles containing HML-2 elements, researchers can gain insights into how these viruses may contribute to disease development and progression. Finally, by better understanding the mechanisms underlying HML-2-induced diseases, researchers can develop more effective treatments targeting specific aspects of viral replication or transmission. 

The findings of Morozov et al. on HERV-K (HML-2) from teratocarcinoma cell lines hold promise in understanding the mechanisms underlying HERV-K (HML-2)-induced diseases and discovering enhanced treatment approaches. A comprehensive analysis of HML-2 parameters and properties, including structural proteins, viral RNA, and particle morphology, reveals the presence of truncated forms of transmembrane proteins. Furthermore, electron microscopy uncovers the existence of free virions and microvesicles carrying HML-2 elements, capable of eliciting specific immune responses and modulating recipient cells. Sequencing findings highlight distinct HERV-K108 env presence in Tera-1 cells and multiple env sequences in GH cells [174]. The research suggests that the viral cargo delivered by microvesicles containing HML-2 elements can induce prominent immunomodulation in recipient cells, possibly contributing to developing diseases. The authors propose that the entire HML-2 particles or fragments might be captured during the shedding of microvesicles, which allows for delivering viral cargo to recipient cells. This “forced cooperation” with microvesicles permits the translocation of HML-2 and its potential pathogenic effects to other cells in the body. While the mechanisms underlying HML-2-induced diseases are not fully understood, this study provides valuable insights into how these viruses may contribute to disease development and progression.

In vitro studies on HERV-K (HML-2) from teratocarcinoma cell lines provide insights into virus assembly and transmission [175,176]. However, further research is necessary to validate therapeutic targets and develop effective antiviral therapies. While teratocarcinoma cell lines may not fully represent normal cellular processes, caution is needed when generalizing findings. The applicability of sucrose density gradient centrifugation for virus purification may be limited to specific samples. Therefore, in vitro studies alone may not identify effective antivirals or fully elucidate the mechanism of HERV-K (HML-2) action [177].

Despite limitations, this study offers significant contributions. It comprehensively analyzes HERV-K (HML-2) from teratocarcinoma cell lines, improving our understanding of virus assembly and transmission. Detecting viral cargo in microvesicles provides insights into their role in disease development. We should continue to explore this connection. 

In silico studies have emerged as a valuable tool in biomedical research, offering a cost-effective and time-efficient approach to exploring various aspects of disease mechanisms and treatment strategies. One area where in silico studies have made significant contributions is in the prediction of efficacy and dose-response of antivirals. Thus, we could bring this to the context of exploring HERV-K (HML-2) mechanisms in cell lines and microvesicles. In silico studies utilize computational models and algorithms to simulate and predict the behavior of biological systems. By leveraging existing knowledge and experimental data, these studies provide insights into the interactions between antiviral compounds and HERV-K, enabling the prediction of drug efficacy and dose-response relationships. This approach offers a cost-effective and efficient alternative to traditional experimental methods, as it reduces the need for extensive laboratory testing. Furthermore, in silico studies can unravel disease mechanisms associated with HERV-K by investigating the molecular interactions between the retrovirus and host cells, simulating the complex biological processes involved in HERV-K activation, replication, and pathogenicity, and identifying critical molecular targets for therapeutic intervention. 

Inspired by Karlsson et al., we identified a potential approach to explore new treatments using nonlinear mixed-effects modeling for HERV-K (HML-2). This approach can account for cell-to-cell variation in parameter estimation using a hierarchical model that includes fixed and random effects. The fixed effects represent the average behavior of all cells, while the random effects capture the variability between individual cells. Nonlinear mixed-effects modeling has already been applied in real-world single-cell studies. One example is a study on fluorescence recovery after photobleaching (FRAP), where NLME was used to estimate parameters related to protein mobility and binding kinetics in individual cells [178,179]. Another example is a study on gene expression noise, where NLME was used to estimate parameters related to transcriptional bursting and degradation rates in single cells. The study used a standard model of gene expression for yeast cells. Parameters for individual cells were estimated using high-quality single-cell measurements of the response of yeast cells to repeated hyperosmotic shocks and state-of-the-art statistical inference approaches for mixed-effects models [98]. This study implies that parameters of models of intracellular processes should be fitted to individual cells to obtain a population of models of similar but non-identical individuals, which can help understand individual cell identity. By employing NLME to analyze single-cell data and quantitatively describe the interactions between antiviral drugs and cells expressing HERV-K (HML-2), we could identify covariates that influence the drug response and viral cargo variation in this specific population of cells—considering factors such as drug concentration, cell proliferation, viability, and protein concentrations.

Based on the data acquired on the prediction of the effect of different antivirals and dose regimens on the population, we can extrapolate the findings to predict the drug response of different antivirals on the population level. This will involve relating the active drug concentration to in vivo dose equivalents, building physiologically based pharmacokinetic (PBPK) models, and validating the predictions against realistic exposure estimates derived from biomonitoring data.

Based on these examples, this approach is allied to other in silico tools for predicting the efficacy and dose-response of antivirals on HERV-K (HML-2) viral cargo. Key methods and techniques used in in silico studies for predicting the impact of antivirals on HERV-K viral cargo are as follows: (1) bioinformatics and genomic analysis, which play a crucial role in understanding the structure and function of HERV-K elements. By utilizing genomic databases and bioinformatics tools, researchers can identify potential drug targets within the HERV-K genome; (2) virtual screening to identify potential drug candidates that can interact with specific viral targets. Using in silico approaches, researchers can screen large libraries of chemical compounds to predict their potential binding affinity and inhibitory effects on viral components. Virtual screening techniques, such as molecular docking and structure-based drug design, enable the identification of promising antiviral candidates that can selectively target HERV-K viral cargo; (3) molecular docking simulations are designed to predict the binding affinity and interaction between antiviral drugs and viral targets. This technique involves computationally modeling the three-dimensional structures of drug molecules and viral proteins or enzymes to assess the likelihood of effective drug-target binding; (4) systems biology modeling, which integrates experimental data and computational algorithms to simulate the dynamics of HERV-K viral cargo and its response to antiviral drug treatments. By developing mathematical models, researchers can predict the impact of drug interventions on HERV-K expression and replication. These models consider various factors such as drug pharmacokinetics, viral kinetics, and host-virus interactions to provide insights into the efficacy and dose-response relationships of antiviral drugs; (5) network analysis approaches can be applied to investigate the interactions between HERV-K elements and cellular components. By constructing and analyzing interaction networks, researchers can identify key nodes or pathways involved in HERV-K expression and assess the potential effects of antiviral drugs on these networks. Network analysis provides a systems-level understanding of the impact of antiviral interventions on HERV-K viral cargo and can guide the identification of critical targets for drug development.

Additionally, an essential aspect to consider in bioinformatics methods is the precise reference of HERVs employed. Regardless of the method employed, it is crucial to specify the genomic references and locations of HERVs under investigation. This information is critical for ensuring accurate analysis and interpretation of HERV-related data. Therefore, it is pertinent to discuss the challenge of multimapping, a common issue encountered when dealing with repetitive elements such as HERVs. Multimapping occurs when sequence reads can be mapped to multiple locations in the genome due to sequence similarity, making it challenging to determine the true source of the read. To address multimapping concerns, recent advancements in bioinformatics tools have provided valuable solutions. Notably, Telescope, a computational software tool described in a study by Bendall et al., offers accurate estimation of transposable element expression, including HERVs, with resolution to specific genomic locations. Telescope tackles the issue of fragment assignment uncertainty by employing a Bayesian statistical model to reassign ambiguously mapped fragments to their most probable source transcript. This approach has been demonstrated to be highly effective in characterizing HERV expression within the transcriptomic landscape. The study by Bendall et al. employed Telescope for single locus analysis of HERV expression in various cell types and revealed significant differences in the retrotranscriptome across these cell types [180]. Telescope’s resolution extends to specific TE insertions, providing a more precise estimation of expression compared to methods that quantify TE expression at broader subfamily levels. In conclusion, specifying the genomic reference of HERVs and addressing the challenge of multimapping using advanced bioinformatics tools like Telescope are crucial steps in HERV-related research. These approaches enhance the accuracy and reliability of HERV expression analysis and contribute to a deeper understanding of their roles in various biological contexts.

## 5. Conclusions

The correlation between aberrant HERV-K (HML-2) expression and various diseases suggests its potential as a diagnostic or prognostic biomarker. However, further research is required to establish HERV-K (HML-2) as a causative factor and to identify specific HERV loci associated with particular diseases. Methodological limitations have hindered studies on the role of specific HERV-K (HML-2) loci. Nonetheless, advancements in sequencing methods and bioinformatic tools offer promising prospects for understanding the causation and development of diseases. The literature reviewed strongly suggests that viral infections can trigger HERV-K (HML-2) reactivation and expression, potentially exacerbating disease progression or influencing the immune response. However, this interaction’s exact mechanisms and implications remain to be fully elucidated. Moreover, the active involvement of HML-2 reactivation in tumor progression indicates that it is more than a mere byproduct of carcinogenesis. HML-2 expression is consistently correlated with disease progression and poor outcomes in various tumors. While larger cohort validations are needed, HML-2 mRNA/protein expression and autoantibodies show potential as robust tools for molecular diagnostics. Functionally, HML-2 proteins contribute to multiple cancer hallmarks, including tumor growth, metastasis, immune evasion, and tumor-promoting inflammation, making them attractive targets for personalized treatment of aggressive tumor subsets with high expression. 

However, challenges remain in HML-2 biology. Poor annotation in human genome assemblies and the repetitive nature of HML-2 transcripts pose obstacles to conventional RNA-seq and bioinformatic analyses. Accurate quantification and representation of HML-2 loci are essential. The lack of comprehensive information on the tertiary structure and functional motifs of HML-2 proteins limits our understanding of their interactions and potential for drug discovery. Moreover, an extensive catalog of TE/HERV transcript and protein expression in healthy and diseased tissues is necessary to definitively unravel their association with diseases and determine their physiological role.

Furthermore, as HML-2 mRNAs can stimulate innate immune receptors, efforts should be made to differentiate their effects from their protein products. Overcoming these challenges and advancing our knowledge of HML-2 biology will facilitate the development of improved diagnostic tools, targeted therapies, and a deeper understanding of their contribution to disease processes.

## Figures and Tables

**Table 2:** Information about the suppression mechanism of HERVs to better understand the development of disease [72].

Suppression Mechanism	Description	Outcome	Example
DNA Methylation	Chemical modification of DNA by adding a methyl group to cytosine nucleotides. Methylation of ERV sequences, especially regulatory regions, prevents gene expression by blocking access to the transcription machinery.	Suppression of ERV expression and potential protection against harmful effects of ERV activation.	Loss of DNA methylation in ERVs can lead to their aberrant activation, resulting in genomic instability and increased risk of diseases like cancer (e.g., hypomethylation-induced activation of oncogenic ERVs in certain cancers).
Histone Modification	Modification of histone proteins, which DNA coils around, affecting gene expression. Histone methylation and deacetylation near ERVs maintain a repressive chromatin structure, inhibiting access to ERV sequences by the transcription machinery.	Silencing of ERVs and prevention of their transcriptional activity.	Dysregulation of histone modifications can lead to the reactivation of ERVs and contribute to various diseases, including autoimmune disorders (e.g., abnormal histone modifications disrupting ERV silencing and triggering autoimmunity in systemic lupus erythematosus).
piRNA Pathway	It involves the production of piRNAs, small non-coding RNA molecules that bind to Piwi proteins. piRNA-Piwi complexes recognize and target ERV transcripts or DNA copies, leading to their degradation and preventing expression.	Suppression of ERV activity by degradation of ERV transcripts or DNA copies.	Dysfunction in the piRNA pathway can result in the derepression of ERVs and contribute to developmental abnormalities and diseases like infertility (e.g., mutations in piRNA pathway components leading to loss of ERV silencing and germ cell defects).
RNA Interference (RNAi)	Small interfering RNAs (siRNAs) or microRNAs (miRNAs) derived from ERV transcripts can trigger RNAi-mediated degradation of complementary ERV RNA molecules, inhibiting their expression.	Degradation and inhibition of ERV transcripts prevent their expression.	Impairment of RNAi machinery can disrupt ERV silencing and potentially contribute to neurodegenerative diseases (e.g., dysregulation of RNAi allowing aberrant expression of neurotoxic ERVs in disorders like amyotrophic lateral sclerosis).
Transcriptional Repressors	Transcription factors and other proteins bind to specific DNA sequences to repress ERV transcription. They interfere with transcriptional activators or recruit additional factors to establish a repressive chromatin environment.	Repression of ERV transcription and prevention of their activation.	Disruption of transcriptional repressor-mediated suppression can lead to the reactivation of ERVs and contribute to diseases like cancer (e.g., loss of transcriptional repressor binding resulting in aberrant ERV expression and oncogenic transformation).
Antiviral Defense Pathways	Activation of antiviral defense pathways, such as interferon signaling, can induce an immune response against ERVs. The immune response produces factors that interfere with viral replication and transcription, suppressing ERV expression.	Suppression of ERV replication and transcription through immune-mediated mechanisms.	Dysregulation or failure of antiviral defense pathways can contribute to ERV activation and associated pathologies, including autoimmune diseases (e.g., deficiencies in antiviral defenses leading to ERV activation and autoimmune responses in Aicardi–Goutières syndrome).

**Table 3:** Summary of the different viruses that are implicated with HERV-K.

The Virus Implicated with HERV-K (HML-2)	Summary
HIV-1	HIV-1 infection activates HERV-K (HML-2) loci, particularly LTR12C repeats associated with antiviral immunity. HERV-K (HML-2) upregulation associated with immune dysregulation, inflammation, and involvement in HIV pathogenesis.
COVID-19	High expression of HERV-K (HML-2) observed in COVID-19 patients, linked to interferon secretion.
HTLV-1	HTLV-1 infection induces the expression of HERV antigens, leading to the activation of HERV-specific T-cell responses.
HCV-infected patients with liver cirrhosis	Increased levels of HERV-K (HML-2) transcripts in HCV-infected patients with liver cirrhosis, indicating a correlation between HERV expression and reduced liver function.
EBV	Increased expression of HERV-K loci and other genes associated with relapses in MS.

**Table 5:** List of antiviral drugs studied in HERV-K (HML-2). Drugs with no reference, our research found no study linking both the drug and HERV-K (HML-2). (NA—Not Applicable).

Class	Possible Mechanism of Action in HERV-K (HML-2)	Drug	References
Non-Nucleoside Reverse Transcriptase Inhibitor	HERV-K (HML-2) also encodes the reverse transcriptase enzyme, which is involved in the conversion of its RNA genome into DNA for integration into the human genome. Therefore, NNRTIs could inhibit the reverse transcription process of HERV-K (HML-2), leading to reduced viral DNA synthesis and integration.	Etravirine	[11]
		Nevirapine	[143]
		Cabovir	[144]
		Efavirenz	[143]
		Cabotegravir	NA
		Delavirdine	NA
		Doravirine	NA
		Rilpivirine	NA
		Abacavir	[138]
		Lamivudine	NA
		Zidovudine	[145]
		Didanosine	NA
		Emtricitabine	NA
		Stavudine	[143]
		Tenofovir disoproxil fumurate	NA
Protease Inhibitors	HERV-K (HML-2) also encodes protease enzymes, and inhibiting this protease could interfere with the proper maturation of HERV-K (HML-2) viral particles. This could produce noninfectious HERV-K (HML-2) particles, reducing their ability to spread and infect new cells.	Ritonavir	NA
		Atazanavir	NA
		Darunavir	[146]
		Fosamprenavir	NA
		Indinavir	NA
		Lopinavir	[146]
		Nelfinavir	NA
		Ritonavir	NA
		Saquinavir	NA
		Tipranavir	NA
Integrase Inhibitors	HERV-K (HML-2) relies on its integrase enzyme to integrate its DNA into the human genome. Integrase inhibitors could block this step, preventing the stable integration of HERV-K (HML-2) DNA into the host cell genome. This could lead to incomplete replication and reduced viral spread.	Elvitegravir	NA
		Dolutegravir	[138]
		Bictegravir	NA
		Cabotegravir	NA
		Raltegravir	[143]

**Table 6:** ClinicalTrials.gov search results with the term HERV-K.

NCT Number	Title	Status	Condition	Intervention	Characteristics	Population
NCT02437110	HERV-K Suppression Using Antiretroviral Therapy in Volunteers With Amyotrophic Lateral Sclerosis (ALS)	Active, not Recruiting	Amyotrophic Lateral Sclerosis	Drug: Darunavir Ritonavir Dolutegravir Tenofovir alafenamide (TAF)	Study Type: Interventional Phase: Phase 1 Primary Purpose: Treatment	Enrollment: 122 Age: 18 Years and older (Adult, Older Adult) Sex: All
NCT01528865	Safety and Efficacy of Lamivudine and Tenofovir to Lower Plasma Level of Viral RNA in Lymphoma	Withdrawn	Lymphoma	Drug: Lamivudine Drug: Tenofovir disoproxil fumarate	Study Type: Interventional Phase: Phase 1 Phase 2 Primary Purpose: Viral load and tumor regression	Enrollment: 0 Age: 18 Years and older (Adult, Older Adult) Sex: All
NCT02171884	Study of the Impact of Freezing/Thawing Procedure and the Prolonged Culture of Embryos on Epigenetic Regulation in Humans	Recruiting	Assisted Reproductive Technology Freezing/Thawing Procedure and Prolonged Culture Procedure	Other: Sample of cord blood Other: Sample of placenta	Study Type: Observational Study Design: Observational Model: Cohort Time Perspective: Prospective Outcome	Enrollment: 366 Age: 18 Years to 50 Years (Adult) Sex: All
NCT05193994	Triumeq in Amyotrophic Lateral Sclerosis (LIGHTHOUSE II)	Recruiting	Amyotrophic Lateral Sclerosis	Drug: Dolutegravir, Abacavir and Lamivudine Drug: Placebo	Study Type: Interventional Phase: Phase 3 Study Design: Allocation: Randomized Intervention Primary Purpose: Treatment Outcome	Enrollment: 390 Age: 18 Years and older (Adult, Older Adult) Sex: Al

## Data Availability

Not applicable.
